# Identification of the Facial Features of Patients With Cancer: A Deep Learning–Based Pilot Study

**DOI:** 10.2196/17234

**Published:** 2020-04-29

**Authors:** Bin Liang, Na Yang, Guosheng He, Peng Huang, Yong Yang

**Affiliations:** 1 Department of Radiation Oncology National Cancer Center/National Clinical Research Center for Cancer/Cancer Hospital Chinese Academy of Medical Sciences and Peking Union Medical College Beijing China; 2 South Building #2 Division The 3rd Medical Center of the People's Liberation Army General Hospital Beijing China; 3 People's Hospital of Beijing Daxing District Beijing China

**Keywords:** convolutional neural network, facial features, cancer patient, deep learning, cancer

## Abstract

**Background:**

Cancer has become the second leading cause of death globally. Most cancer cases are due to genetic mutations, which affect metabolism and result in facial changes.

**Objective:**

In this study, we aimed to identify the facial features of patients with cancer using the deep learning technique.

**Methods:**

Images of faces of patients with cancer were collected to build the cancer face image data set. A face image data set of people without cancer was built by randomly selecting images from the publicly available MegaAge data set according to the sex and age distribution of the cancer face image data set. Each face image was preprocessed to obtain an upright centered face chip, following which the background was filtered out to exclude the effects of nonrelative factors. A residual neural network was constructed to classify cancer and noncancer cases. Transfer learning, minibatches, few epochs, L2 regulation, and random dropout training strategies were used to prevent overfitting. Moreover, guided gradient-weighted class activation mapping was used to reveal the relevant features.

**Results:**

A total of 8124 face images of patients with cancer (men: n=3851, 47.4%; women: n=4273, 52.6%) were collected from January 2018 to January 2019. The ages of the patients ranged from 1 year to 70 years (median age 52 years). The average faces of both male and female patients with cancer displayed more obvious facial adiposity than the average faces of people without cancer, which was supported by a landmark comparison. When testing the data set, the training process was terminated after 5 epochs. The area under the receiver operating characteristic curve was 0.94, and the accuracy rate was 0.82. The main relative feature of cancer cases was facial skin, while the relative features of noncancer cases were extracted from the complementary face region.

**Conclusions:**

In this study, we built a face data set of patients with cancer and constructed a deep learning model to classify the faces of people with and those without cancer. We found that facial skin and adiposity were closely related to the presence of cancer.

## Introduction

In daily social activities, people tend to judge the health status of others based on their facial appearance. Facial appearance is associated with other social judgments, such as leadership ability and attractiveness. These judgments may affect real-life outcomes such as employment and dating outcomes. Numerous studies have indicated that healthy people tend to be perceived as more attractive [1-4]. This may be explained by Darwin’s theory of evolution: This preference has evolved because these “attractive” traits provide signals of biological quality, particularly physical health, and keeping away from individuals who are perceived to be less attractive (unhealthy) may prevent the contraction of a contagious disease.

Most published research focuses on the association of health status with perceived features such as skin color, facial adiposity, and symmetry. Skin color is malleable and may change rapidly in response to health status. Thus, it provides more relevant information regarding an individual’s current physical condition [5-10]. Facial adiposity is positively correlated with BMI and body fat percentage, and it conveys important information about one’s physical condition [1,11]. Facial symmetry is considered to be an indicator of health because it corresponds with the ability to maintain developmental stability and resist asymmetric growth (eg, pathogens or mutation rate) [4,12-15]. These studies attempt to reveal the association of perceptual facial features with physical condition, which is helpful in understanding the underlying psychological and biological reasons for an individual’s behavior and social activities.

The recently developed deep learning (DL) technique [16] provides a novel method to study the correlation of facial features with health status. The facial features are extracted via a large-scale cascaded conventional neural network (CNN), which is more subjective than perceptual features. Gurovich et al [17] adopted the DL technique to identify facial phenotypes of genetic disorders. Kong et al [18] reported detection of acromegaly using the DL technique based on facial images; they achieved satisfactory accuracy and sensitivity. Wang et al [19] found that the DL technique outperformed humans at detecting sexual orientation based on facial images.

Cancer caused approximately 9.6 million deaths worldwide in 2018 [20]. It has become the second leading cause of death globally. Approximately 90%-95% of cancer cases are due to genetic mutations caused by environmental (radiation, pollution, etc.) and lifestyle (tobacco, alcohol, etc.) factors, while the remaining cases are due to inherited genetics. In either case, genetic mutation may result in facial features that are different from those of people without cancer. In this study, we aimed to reveal the facial features of patients with cancer using DL techniques and to use these features to distinguish the faces of people with cancer from those of people without cancer.

## Methods

In this section, the face image data sets for people with and those without cancer are briefly introduced. Subsequently, the preprocessing procedure and network architecture are presented in detail. Finally, the transfer learning strategy and training details are described.

### Ethics Statement

This study was carried out in accordance with the Declaration of Helsinki and approved and exempted from requiring informed consent by the Independent Ethics Committee of the Cancer Hospital of the Chinese Academy of Medical Sciences.

### Image Data Sets

Images of the faces of patients with cancer at our institution before radiotherapy were collected over 1 year to build the cancer face image data set. The ages of the patients with cancer ranged from 1 year to 70 years, and the median age was 52. The histogram of the age distribution is shown in Table 1. The noncancer face image data set was built by randomly sampling the MegaAge data set [21]. The MegaAge data set consists of 41,941 faces of Asian people with annotated ages. Considering data balance, 8124 face images with the same sex ratio and age distribution as the cancer face data set were randomly selected from the MegaAge data set.

**Table 1:** Age distribution of the patients in the cancer face data set.

Age category	Patients	
	Male, n (%)	Female, n (%)
<10 years	31 (0.80)	28 (0.66)
10-20 years	41 (1.06)	23 (0.54)
20-30 years	81 (2.10)	163 (3.81)
30-40 years	258 (6.70)	738 (17.27)
40-50 years	689 (17.89)	1542 (36.09)
50-60 years	1558 (40.46)	1375 (32.18)
60-70 years	1193 (30.98)	404 (9.45)

### Preprocessing

**Figure 1:**
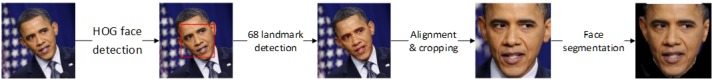
Pre-processing workflow to obtain chip images to use as inputs in the convolutional neutral network, showing the intermediate results. Image courtesy Barack Obama Presidential Library [22].

To exclude the influence of nonrelevant factors such as face pose, body profile, and image background, only upright, centered, and frontal face “chip” images were used as inputs of the convolutional neutral network (CNN). The preprocessing workflow and the intermediate results are shown in Figure 1 [22]. The human face in each image was first detected based on the histogram of oriented gradients (HOG) [23]. The positions of 68 face landmarks such as the corners of the mouth and the centers of the eyes were then detected [24]. Using these landmarks, the face pose was determined. The image was rotated and aligned accordingly to obtain an upright, frontal, centered face image. The aligned image was cropped to obtain the face chip (ie, only the face part was retained and any other parts of the image were removed). Finally, the background in the resulting chip was further filtered out. The human face in the image was segmented using a fully convolutional network (FCN) [25,26], and the background was masked.

### Network Architecture

**Figure 2:**
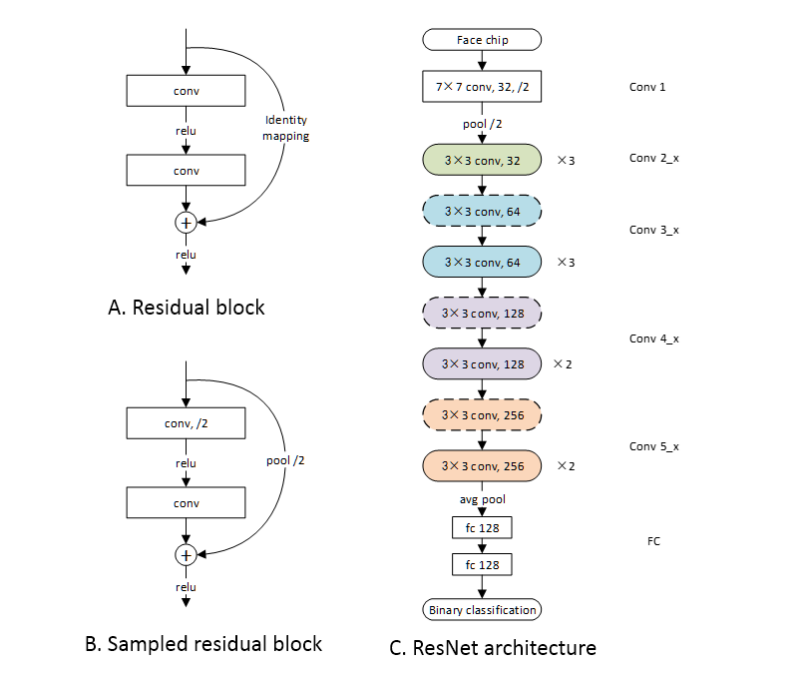
Network architecture. A) The basic cell of ResNet: the residual block. B) The sampled residual block. The difference is that the convolution of the first layer is performed with a 2-pixel stride. Correspondingly, 2-pixel stride sampling was performed before identity mapping. C) The ResNet architecture. The blocks shown in A) and B) are annotated with solid and dashed round-ended boxes, respectively.

In this work, a residual neural network (ResNet) was used to extract facial features. ResNet was proposed in 2015 by He et al [27]. Because a CNN is trained based on the gradient of the loss function, the gradient of the cascaded network becomes smaller as the network deepens. This issue was solved by adding identity shortcut connections to skip the convolutional layers in between, as shown in Figure 2A and B. The network of this study contains 27 convolutional layers and 2 full connection (FC) layers. The architecture is shown in Figure 2C, and the number and sizes of the filters in each layer are also annotated. Essentially, the model is a simplified version of ResNet-34 implemented proposed in the paper by He et al [27], with 7 layers removed and the number of filters in each layer reduced by half.

### Transfer Learning and Training Strategy

For the convolution layers (layers 1-5), the weights of a pretrained face encoding network [28] were directly utilized. The face-encoding network contains the same convolution layers but only one FC layer. With this structure, the features extracted by the convolution layers are flattened into a one-dimensional vector by the FC layer. In other words, the face image is encoded into a one-dimensional vector. The Euclidean distance between two vectors directly measures the similarity of the two corresponding faces. With the triplet loss function, the encoded vectors of the face images of the same individual are mapped close to each other in the Euclidean space, while the vectors of different individuals are mapped far from each other. Through this process, the encoding network captures the features that are unique to an individual.

This study aimed to determine what the faces of patients with cancer have in common from these individually distinguishable features. For our model, the weights of the convolution layers were fixed, and only the weights of the FC layers were trained for classification. This is because the weights of the convolution layers were trained on a combined data set with about 3 million faces [29,30], which is several orders of magnitude larger than the size of our data set. Addition of our data set would not introduce any substantial improvements.

In the data set, 50% of the face chips were randomly selected for training and 50% were selected for testing. To reduce the risk of overfitting, the following strategies were adopted: The minibatch size was set to 10, only 5 epochs were used, an L2 regulation penalty was added to the weights of the FC layers, and a random dropout was added on the FC1 layer. Both the learning rate and weight of the L2 regulation terms were set to 0.001. The dropout rate was set to 0.25; therefore, 25% of the extracted features were randomly dropped. The guided gradient-weighted class activation mapping (grad-CAM) method was utilized in the trained model to illustrate the discriminative features [31] of the cancer and non-cancer data sets. The guided grad-CAM method is the Hadamard production of global backpropagation and CAM. The global backpropagation is the gradient of the network, and the CAM is the activated map of each class.

This study was implemented with Python programming language. All data preprocessing procedures except FCN face segmentation were implemented using the Dlib package (v19.17.0). FCN face segmentation and ResNet were implemented using TensorFlow (v1.4.0) [32] and Keras (v2.2.4).

## Results

We collected images of the faces of 8124 patients with cancer before radiotherapy from January 2018 to January 2019, including 3851 (47.4%) men and 4273 (52.6%) women. We compared the average faces of the cancer and noncancer data sets and presented the training and validation results; finally, we showed the distinguishable facial features of people with and those without cancer, captured by the grad-CAM method.

### Average Faces

**Figure 3:**
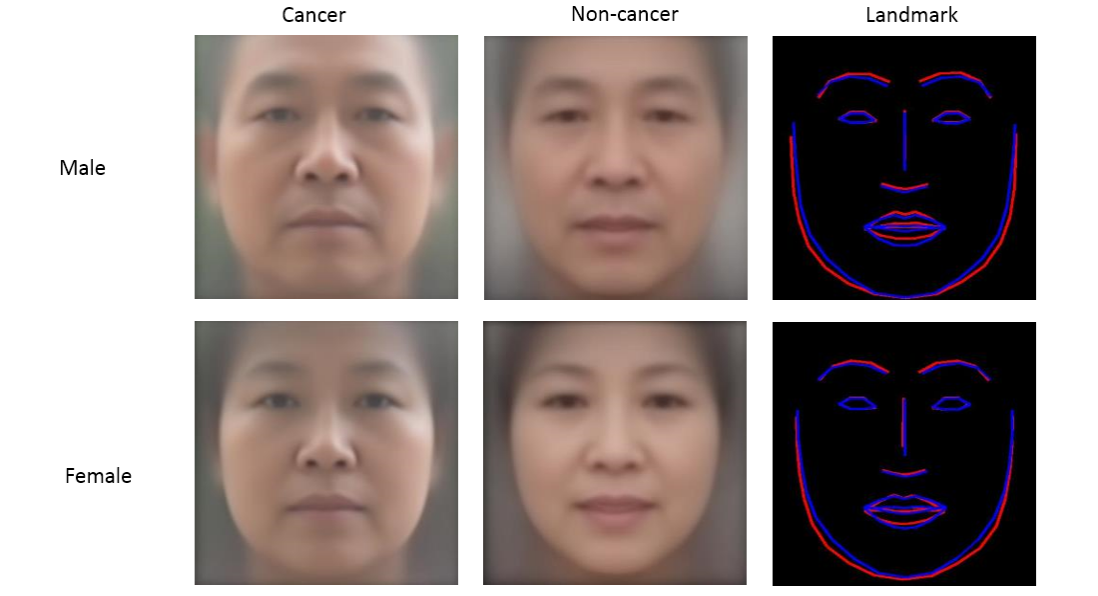
Average faces and landmark comparison. Top row: male, bottom row: female. First column: cancer, second column: noncancer, and third column: landmark comparison (from left to right). In the third column, the landmark of the average cancer face is depicted in red and that of the average noncancer face is depicted in blue.

The intermediate results of the preprocessing procedure are shown in Figure 1. The average faces are shown in Figure 3. The facial landmarks of the average cancer face and the average noncancer face were detected and are depicted in the same figure for comparison. Intuitively, both the male and female average cancer faces display more obvious facial adiposity than the average noncancer faces, which was supported by the landmark comparison.

### Training and Testing Results

**Figure 4:**
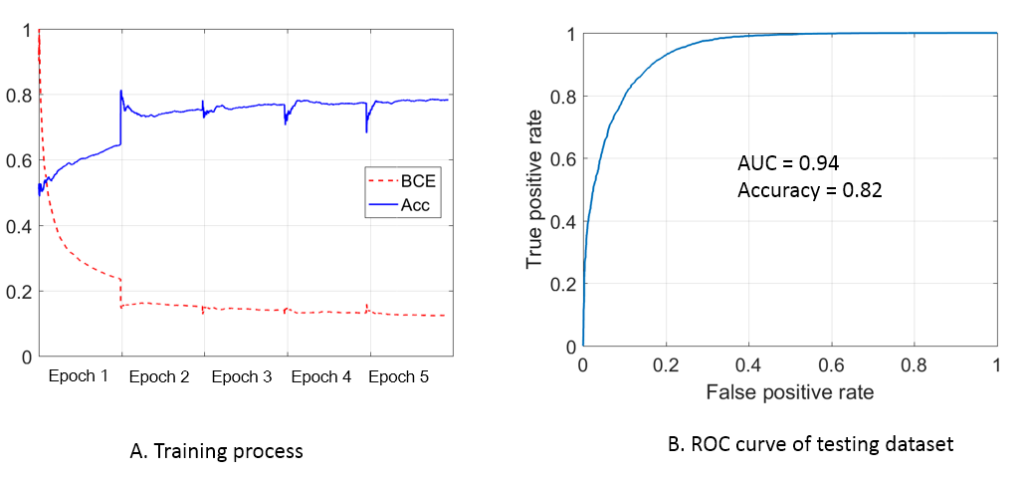
Training and testing results: (A) Binary cross-entropy (BCE) and accuracy (Acc) during the training process. The loss value is normalized for clarity. (B) Receiver operating characteristic (ROC) curve of the testing dataset (AUC: area under the curve).

The binary cross-entropy (BCE) and accuracy during the training process are shown in Figure 4A. The fluctuation at the beginning of each epoch is due to the random shuffle of the training batch. For both BCE and accuracy, the most significant improvement was observed in epoch 1, and the improvement decreased with each epoch. No obvious improvement was observed between epoch 4 and epoch 5. The training process was terminated after 5 epochs to reduce the risk of overfitting. The receiver operating characteristic (ROC) curve of the testing data set is shown in Figure 4B. The accuracy value is 0.82, which is consistent with the training results (approximately 0.80). The area under the curve (AUC) is 0.94. The ROC curve is calculated with various “passing thresholds,” and the corresponding AUC measures the discriminative ability of the model. The accuracy evaluates the discriminative ability with the “passing threshold” fixed to 0.5.

### Guided Grad-CAM

**Figure 5:**
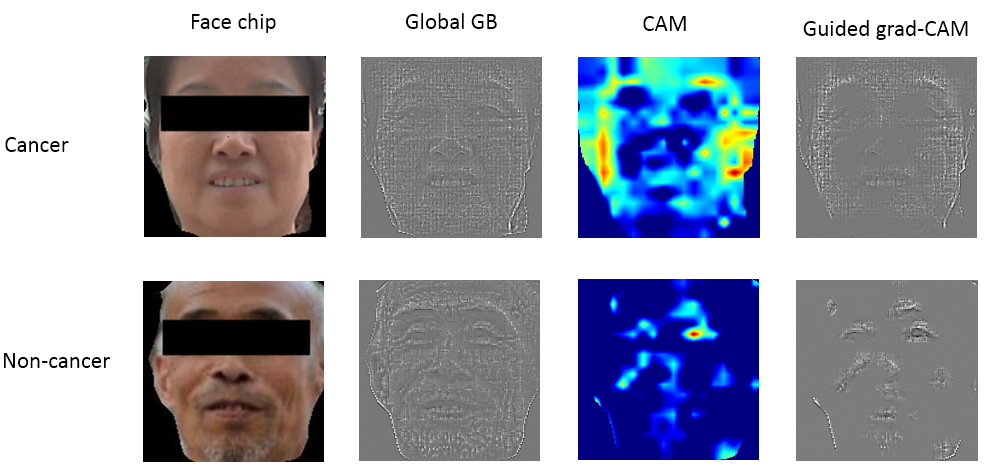
Grad-CAM analysis results for face images of people with and without cancer.

Figure 5 shows the results of the guided grad-CAM method for a typical cancer case and a typical noncancer case. The global backpropagation can be interpreted as the features extracted by the convolutional network. As shown in Figure 5, the global backpropagations of the two cases show the same pattern. This is because during the convolution process, “complete” features were extracted and the relative features of each class were activated by the following FC layers. The relative features of each class are shown by the guided grad-CAM, which is the entry-wise product of the global backpropagation and CAM. The main relative feature of people with cancer is the facial skin, that is, the facial region except for the eyes, eyebrows, lips, and nose. In contrast, the relative features of patients without cancer only include features extracted from the eyes, lips, and end-of-nose regions. This finding supports the findings from the average faces, where the average cancer face showed more obvious facial adiposity.

## Discussion

In this study, we built a face data set of patients with cancer and constructed a deep learning model to classify the faces of people with and those without cancer. We found that facial skin and facial adiposity were closely related to the presence of cancer.

The AUC and accuracy results indicate that the network can discriminate the faces of patients with cancer from those of people without cancer. External validation must be performed to improve the presented network. Although facial features are closely related to an individual’s health status, it is not solid to predict cancer incidence based on facial features. The motivation of this study was to find the facial features of cancer patients. The MegaAge data set was used as a noncancer reference data set. The possibility of people with cancer in the MegaAge data set cannot be excluded. However, the incidence of cancer cases in China is only 0.3%-0.4% [33], which would not affect the results.

We mainly attribute the satisfactory AUC and accuracy results to the transfer learning strategy. The parameters of the deep network were pretrained well with a large data set. We would like to point out that the convolution network was designed and pretrained for face image–based individual recognition, and only the FC layers were trained for classification in this study. The results demonstrate that cancer-discriminative facial features were included in the individual-discriminative features. The preprocessing procedure, which excludes the effects of factors other than the face chip, is another reason for the satisfactory results.

This study reveals that facial skin and adiposity are closely related to the presence of cancer. It has been proven that facial skin and adiposity are associated with health status. Among all facial features, these two respond most rapidly to the metabolic status. This finding can possibly be explained by the fact that malignant tumors affect metabolism and thus further affect facial skin and adiposity.

## Abbreviations

AUC: area under the curve
BCE: binary cross-entropy
CAM: class activation mapping
CNN: conventional neural network
DL: deep learning
FC: full connection
FCN: fully convolutional network
grad-CAM: gradient-weighted class activation mapping
HOG: histogram of oriented gradients
ResNet: residual neural network
ROC: receiver operating characteristic
